# The Evolution of Proximal Femur Hemiepiphysiodesis in Children with Cerebral Palsy

**DOI:** 10.3390/jcm15156057

**Published:** 2026-08-04

**Authors:** Trevor Blanchard, James J. McCarthy, Verena M. Schreiber

**Affiliations:** Division of Orthopaedic Surgery, Cincinnati Children’s Hospital Medical Center, Cincinnati, OH 45229, USA

**Keywords:** cerebral palsy, hip subluxation, guided growth, proximal femur hemiepiphysiodesis

## Abstract

Hip displacement is a common and progressive musculoskeletal complication in children with cerebral palsy (CP), often driven by coxa valga, muscle imbalance, and spasticity. While proximal femoral and pelvic osteotomies remain the standard treatment for advanced deformity, proximal femoral guided growth (temporary medial hemiepiphysiodesis) has emerged as a minimally invasive alternative for selected patients with remaining growth potential. This narrative review summarizes the current evidence regarding indications, surgical technique, radiographic outcomes, complications, and future directions for proximal femoral guided growth in CP. The literature, consisting primarily of retrospective case series and one systematic review, consistently demonstrates improvements in migration percentage, head–shaft angle, and acetabular index, particularly when treatment is performed early in children with migration percentages below 50% and combined with soft tissue release. Younger age and less severe hip displacement are associated with more favorable outcomes. The most common complication is screw migration as the physis grows off the implant, frequently necessitating revision, although serious complications are uncommon. Concerns remain regarding premature physeal closure and potential femoral head deformity at skeletal maturity. Current evidence supports proximal femoral guided growth as a valuable intermediate treatment that may delay or reduce the need for reconstructive osteotomy in appropriately selected patients; however, high-quality prospective studies are needed to define better its long-term safety, efficacy, and optimal role in treatment algorithms.

## 1. Introduction

Hip subluxation represents one of the most common and debilitating musculoskeletal complications affecting children with cerebral palsy, occurring in up to one-third of this patient population (Soo et al., 2006 [1]). The fundamental pathophysiology underlying hip displacement in children with cerebral palsy involves muscle imbalance, spasticity, and abnormal biomechanical forces acting on the developing hip joint. These forces contribute to excessive coxa valga, femoral anteversion, and caput valgum, which promotes lateral migration of the femoral head from the acetabulum [2,3,4,5]. Progressive hip subluxation and dislocation associated with coxa valga deformity have traditionally been managed with extensive reconstructive procedures, including proximal femoral varus osteotomy and pelvic osteotomies with an extensive recovery period [6,7]. Over the past two decades, proximal femoral guided growth, also known as medial hemiepiphysiodesis of the proximal femur, has emerged as a minimally invasive alternative approach to address hip displacement in this vulnerable population.

The concept of guided growth at the proximal femur aims to selectively tether the growth on the medial aspect of the proximal femoral physis, thereby creating a gradual varus correction that improves hip containment and stability (Portinaro et al., 2019 [8]). This approach represents a paradigm shift from immediate surgical correction through osteotomy to a more gradual, growth-modulating strategy that works with the child’s remaining skeletal development.

## 2. Methods

For this narrative review, an extensive review of the literature was performed focusing on proximal femur guided growth in children with cerebral palsy. We utilized the search terms “proximal femur guided growth” and “proximal femur hemiepiphyseodesis”. We then identified papers describing the early research of this procedure, the surgical technique, radiographic and clinical outcome data, and complications of the surgery. Given the relatively new status of this procedure, we did not include any timeline limits for the papers reviewed.

## 3. Early Research

The concept of proximal femur guided growth was initially investigated using animal models [9,10]. McCarthy and colleagues compared this procedure to a sham group (screw that did not cross the physis) in ten 2-month-old lambs [10]. There were no statistically significant differences in the preoperative neck–shaft angles (NSAs) between the intervention and sham groups. They found a decrease of 11 degrees in mean NSA for the intervention group, which was statistically significant. There were no statistically significant changes in the sham group. Similarly, Chang and colleagues performed a similar study in pigs. They found a statistically significant decrease in NSA after a 3-month follow-up [9]. D’Heurle et al. investigated the effect of screw, plate, and drills in asymmetric proximal femoral growth in 8-week-old lambs and concluded that screw implantation appeared to be more effective than plates and drilling [11].

## 4. Patient Selection and Indications

Based on the accumulated evidence, several patient selection criteria and indications for proximal femoral guided growth have emerged from the literature. Age represents an important consideration, with most studies including children between 4 and 12 years of age, though recent evidence suggests potential benefit in children as young as 2 to 4 years. The requirement for at least 2 years of remaining growth is commonly cited, as adequate time is necessary for the guided growth effect to produce meaningful correction. Gross Motor Function Classification System levels have varied across studies, with some focusing on nonambulatory children (GMFCS IV–V) while others have included ambulatory patients (GMFCS I–III), and successful outcomes have been reported across the spectrum of functional abilities.

Radiographic criteria typically include a migration percentage greater than 30% and a head–shaft angle (or neck–shaft angle) greater than 155 degrees, indicating significant coxa valga deformity. The upper threshold for migration percentage remains somewhat controversial, with most evidence suggesting optimal outcomes for migration percentages less than 50%, though some studies have reported success in more severely displaced hips. Acetabular dysplasia, as measured by acetabular index, appears to be an important predictor of outcome, with a higher acetabular index associated with an increased risk of failure. Some authors have suggested that significant acetabular dysplasia may warrant consideration of combined femoral and pelvic procedures rather than isolated proximal femoral guided growth.

The presence of adequate hip abduction is often considered, with some protocols specifying hip abduction of 40 degrees or fewer as an indication for combined soft tissue release. The ability to perform soft tissue release addresses the dynamic component of hip displacement, while guided growth addresses the structural bony deformity. Bilateral involvement is common in cerebral palsy, and the minimally invasive nature of guided growth makes bilateral procedures feasible in a single surgical session.

## 5. Surgical Technique and Technical Considerations

**Figure 1:**
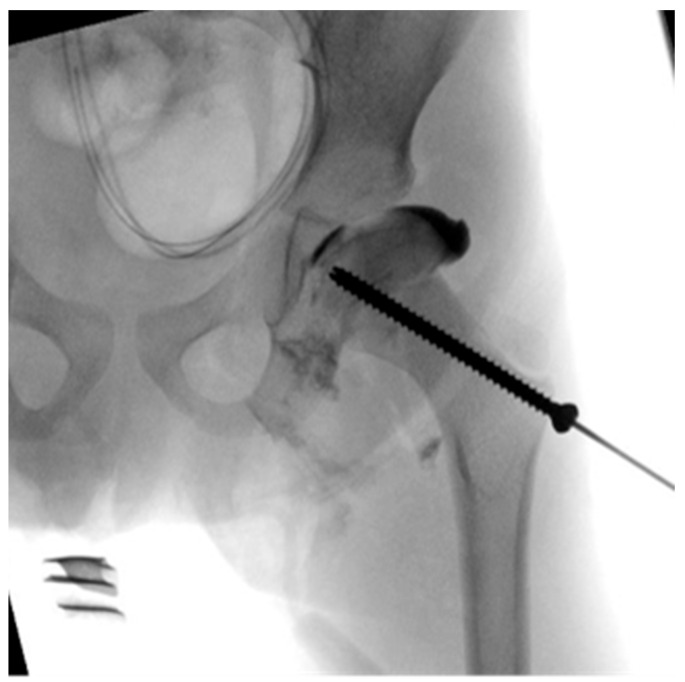
Intraoperative fluoroscopic image of a 5-year-old male with spastic quadriplegic cerebral palsy, GMFCS Level V.

The surgical technique for proximal femoral guided growth involves the percutaneous or minimally invasive placement of a transphyseal screw across the inferomedial portion of the proximal femoral physis under fluoroscopic guidance. The procedure is typically performed with the patient positioned supine, and fluoroscopy is used in both anteroposterior and lateral planes to ensure optimal screw placement. In the anteroposterior view, the screw is inserted across the inferomedial portion of the proximal femoral physis, while in the lateral view, it is centered along the femoral neck. The screw crosses the physis obliquely, creating a tether that selectively inhibits growth on the medial side while allowing continued growth laterally, thereby producing a progressive varus correction over time [12,13] (Figure 1).

The position of the screw within the physis has been demonstrated to significantly influence both the efficacy of correction and the risk of complications. Hsu and colleagues [14] investigated the optimal screw position in a retrospective analysis of 61 hips in 32 children with cerebral palsy who underwent proximal femoral hemiepiphysiodesis. They divided hips into two groups based on transphyseal screw position in the coronal plane: across the medial quarter versus the middle quarter of the medial half of the physis. The study found that more eccentric screw placement (across the medial quarter) resulted in more substantial decreases in head–shaft angle (HSA) and migration percentage but also presented a higher incidence of the physis growing off the screw. Regression analysis indicated that screw eccentricity correlated with greater HSA correction but increased the risk of early revision. The authors recommended a more centered screw position, at least across the middle quarter of the medial physis, particularly for younger children, to balance efficacy with the need to avoid early revision surgery.

The surgical procedure is often combined with soft tissue releases, particularly adductor and psoas tenotomies, to address the underlying muscle imbalance contributing to hip displacement [15]. This combination approach recognizes that guided growth addresses the bony deformity while soft tissue release addresses the dynamic forces contributing to hip instability. The minimally invasive nature of the procedure, with less surgical dissection compared to traditional osteotomies, offers potential advantages including faster recovery of motion, reduced perioperative morbidity, and the ability to perform the procedure bilaterally in a single surgical session when indicated.

## 6. Clinical Outcomes and Radiographic Outcomes

Multiple studies have demonstrated the efficacy of proximal femoral guided growth in improving radiographic parameters of hip displacement in children with cerebral palsy [8,12,13,16,17]. The primary radiographic measurements used to assess outcomes include Reimer’s migration percentage, head–shaft angle (or neck–shaft angle), acetabular index, and Hilgenreiner’s epiphyseal angle. These measurements provide an objective assessment of both the degree of hip subluxation and the severity of coxa valga deformity.

Lee and colleagues reported preliminary results from a case series of 9 children (13 hips) who received soft tissue release and guided growth at the proximal femur, with surgical indications including children with spastic cerebral palsy aged 4 to 10 years, Gross Motor Function Classification System level IV or V, and hip displacement on one or both sides [13]. At a mean follow-up of 45.6 months, the mean migration percentage improved significantly from 52.2% preoperatively to 45.8% at 3 months, 40.3% at 1 year, and 37.1% at 2 years after operation. The head–shaft angle remained unchanged in the first 3 months but decreased from 173.3 degrees to 166.4 degrees at 1 year and to 162.7 degrees at 2 years postoperatively. The authors noted that the immediate postoperative improvement in migration percentage resulted from soft tissue release, while the progressive reduction in head–shaft angle from 3 months to 2 years represented the effect of guided growth. They concluded that less surgical dissection, faster recovery of motion, and less comorbidity compared to varus osteotomy made guided growth surgery a viable treatment option for coxa valga in spastic hip displacement in non-ambulatory children with cerebral palsy.

Hsieh and colleagues conducted a retrospective study of 24 patients (48 hips) who underwent guided growth for progressive bilateral hip subluxation associated with coxa valga deformities between 2012 and 2016 [12]. Surgical indications included a migration percentage greater than 30%, a head–shaft angle greater than 155 degrees, and at least 2 years of growth remaining. At a mean follow-up of 50 months, the study demonstrated significant improvements in both coxa valga and lateral hip subluxation, with a reduction in the head–shaft angle by a mean of 13 degrees and a reduction in the migration percentage by 10%. The study identified that longer follow-up duration and a smaller preoperative migration percentage were associated with larger changes in the head–shaft angle. Importantly, the authors noted that although guided growth did not create as large a degree of varus as proximal femoral osteotomy, it effectively stabilized the hip in children with a migration percentage less than 50%.

Portinaro and colleagues evaluated 28 patients (56 hips) with cerebral palsy and hip dysplasia, aged 4 to 11 years and Gross Motor Function Classification System levels III through V, who underwent temporary medial hemiepiphysiodesis using a transphyseal screw between 2007 and 2010 [8]. Clinical and radiologic evaluation was performed preoperatively and at 6-, 12-, and 60-month intervals following surgery. All radiographic measurements, including acetabular index, neck–shaft angle, and migration percentage, showed significant improvement at the final follow-up. This study concluded that temporary medial hemiepiphysiodesis was effective in controlling progressive subluxation of the hip in the majority of cases, potentially avoiding the need for major reconstructive surgery.

**Figure 2:**
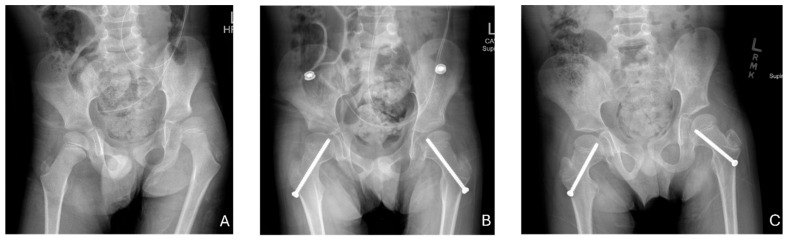
5-year-old male with spastic quadriplegic cerebral palsy, GMFCS Level V, preop (**A**), 15 months postop (**B**), 2 years postop (**C**).

Zakrzewski and colleagues reported on 23 patients (44 hips) who underwent proximal femoral screw hemiepiphysiodesis with at least 2 years of radiographic follow-up [17]. The study divided hips into two groups: those with no previous hip surgery (Group 1, 32 hips) and those where hemiepiphysiodesis was performed after hip reconstruction (Group 2, 12 hips). Group 1 demonstrated significant improvement in all radiographic parameters, with changes in migration percentage of 5%, neck–shaft angle of 13 degrees, and head–shaft angle of 15 degrees. Group 2 showed improvements in neck–shaft angle and head–shaft angle, though only head–shaft angle reached statistical significance, and migration percentage remained unchanged. Importantly, the study included a natural history cohort of 25 hips with 2 years of preoperative radiographic data, demonstrating that all radiographic measurements worsened in the 2 years before surgery but improved after proximal femoral screw hemiepiphysiodesis, providing evidence that the procedure alters the natural history of hip displacement (Figure 2).

Lebe et al. conducted a systematic review and meta-analysis of guided growth of the proximal femur for hips at risk in children with CP. This review included four primary studies encompassing 93 patients and 178 hips [16]. The meta-analysis demonstrated statistically significant improvements in MP decreased by a mean of 8.48%, HSA by 12.28°, and acetabular index by 3.41° at a minimum of two years follow-up. These findings support the efficacy of guided growth in improving hip stability and alignment. However, the authors noted substantial heterogeneity among studies and emphasized the overall low level of evidence, as all included studies were Level IV case series.

## 7. Age-Related Outcomes and Predictors of Success

The relationship between patient age at the time of surgery and treatment outcomes has been an important area of investigation, as younger children have greater remaining growth potential, but may also face unique challenges. Herrero and colleagues specifically evaluated the effects of age and preoperative migration percentage severity on treatment success for young children 6 years of age or younger with cerebral palsy [18]. The study included 44 patients (78 hips) stratified by age (younger than 4 years versus 4 to 6 years) and preoperative migration percentage. All Gross Motor Function Classification System levels showed improvement, with GMFCS I–III improving from a migration percentage of 37% to 29%, and GMFCS IV–V improving from 43% to 37%. Greater migration percentage improvements were observed for hips with a preoperative migration percentage of 40% or greater and for patients younger than 4 years compared to those 4 to 6 years old (41% to 28% versus 37% to 37%, respectively). Definitive improvement, defined as a change in migration percentage of 10% or greater, was seen more frequently in younger children (62% versus 13%), while both age groups demonstrated high success rates in preventing progression (100% versus 81%). The authors suggested that a greater migration percentage improvement with a higher preoperative migration percentage might be secondary to concomitant femoral neck shortening that occurs with the guided growth process.

Similarly, in the article previously reported by Portinaro and colleagues, multiple regression analysis revealed that migration percentage, time from surgery, and age influenced the decrease in neck–shaft angle [8]. Notably, the authors emphasized the importance of early intervention, suggesting that outcomes were more favorable when the procedure was performed before significant hip subluxation had occurred.

Several studies have identified specific predictors of successful outcomes following proximal femoral guided growth. Hsieh and colleagues found that longer follow-up duration and smaller preoperative migration percentage were associated with larger changes in head–shaft angle after controlling for confounding variables including gender, Gross Motor Function Classification System level, Hilgenreiner’s epiphyseal angle, and acetabular index [12]. The study also identified factors associated with treatment failure requiring secondary reconstructive surgery. Specifically, patients who underwent secondary reconstruction had a higher preoperative acetabular index (mean 29 degrees versus 21 degrees), a higher head–shaft angle (mean 166 degrees versus 162 degrees), and a higher migration percentage (mean 54% versus 36%).

The severity of preoperative hip displacement has emerged as a critical factor in determining treatment success. Multiple studies have suggested that guided growth is most effective for hips with a migration percentage less than 50%, with higher failure rates observed in more severely displaced hips. This finding has important implications for patient selection and timing of intervention, suggesting that earlier intervention before severe displacement develops may optimize outcomes. The Gross Motor Function Classification System level has also been identified as a relevant factor, with some studies suggesting higher failure rates in GMFCS IV–V patients compared to GMFCS I–III patients, although successful outcomes have been reported across all functional levels. Finally, age at the time of surgery has been identified as an important predictive factor. Younger age (<4 years) has been correlated with improved success of the procedure.

## 8. Long-Term Outcomes and Skeletal Maturity

While short-term and intermediate-term outcomes of proximal femoral guided growth have been encouraging, concerns about long-term effects on the proximal femoral physis and potential complications at skeletal maturity have prompted investigation. Chiu and colleagues conducted a retrospective study of 29 patients (53 hips) who underwent proximal femur guided growth surgery between 2012 and 2017 and were followed until physeal closure [19]. This study represents one of the first comprehensive long-term analyses examining outcomes at skeletal maturity. Among the cohort, 4 patients (6 hips, 11.3%) experienced procedure failure and required varus osteotomy due to severe deformities, with failure more common in GMFCS IV–V patients (27.3%) compared to GMFCS I–III patients (5.6%). In the remaining 25 patients (47 hips), all radiographic parameters improved significantly. However, the study identified several important concerns related to the procedure. The epiphysis grew off the screw in 25 hips (53.2%), requiring reinsertion in 19 hips (40.4%), with a higher rate in non-ambulatory children (73.3% versus 25%). The cumulative duration of screws crossing the physis was identified as a key factor for earlier physeal closure and correlated with an increased alpha angle, suggesting potential femoral head deformity. The authors concluded that while guided growth successfully improved outcomes in both ambulatory and non-ambulatory groups, the procedure raised concerns including the need for screw reinsertion, earlier physeal closure, and potential femoral head deformity.

The issue of premature physeal closure represents an important consideration, as the goal of guided growth is to modulate rather than completely arrest physeal growth. The finding that the cumulative duration of screw placement correlates with earlier closure suggests that careful monitoring and timely screw removal once adequate correction is achieved may be important to minimize this risk. The development of femoral head deformity, as evidenced by increased alpha angle, raises questions about potential long-term consequences for hip joint health and the risk of early degenerative changes, though longer-term follow-up into adulthood will be necessary to fully understand these implications.

## 9. Complications and Reoperations

The complication profile of proximal femoral guided growth differs substantially from that of traditional osteotomy procedures. The most commonly reported complication is the physis growing off the screw tip, which occurs as the child continues to grow and the epiphysis migrates away from the metaphyseal screw anchor. This phenomenon has been reported in 44% to 53% of hips in various studies, typically occurring at a mean of 28 to 36 months after initial screw placement [8,13,16,19]. While this represents a technical failure of the implant, it is generally managed with a relatively minor revision procedure involving replacement with a longer screw or placement of a second screw parallel to the existing one.

Hsieh and colleagues reported that 31% of hips (15 of 48) in 33% of patients (8 of 24) underwent replacement with a longer screw when the physis grew off the screw tip. Among the 17% of hips (8 of 48) in 21% of patients (5 of 24) who had progressive lateral subluxation requiring secondary reconstructive surgery, these patients had more severe preoperative deformities with higher acetabular index, head–shaft angle, and migration percentage [12]. Zakrzewski and colleagues reported screw exchange in 12 hips (27.2%) at an average of 33 months, with 2 hips also undergoing pelvic osteotomy at that time. Three hips had a migration percentage greater than 50% at follow-up, with 2 hips undergoing hip reconstruction [17].

Portinaro and colleagues reported that the femoral physis grew off the screw in 9 hips within 36 months, with one screw head breaking during an attempted screw exchange. The remaining cases were treated by placing a second screw parallel to the existing one. Progressive subluxation occurred in 3 hips when the physis grew off the screw, and these were treated by skeletal reconstruction [8]. Importantly, across multiple studies, serious complications such as infection, neurovascular injury, or avascular necrosis have been rare or absent, suggesting a favorable safety profile compared to more extensive reconstructive procedures [16].

The need for screw exchange or reinsertion, while common, is generally considered a minor procedure compared to the morbidity associated with revision osteotomy. However, the frequency of this occurrence highlights the importance of careful patient counseling regarding the potential need for additional procedures and the necessity of close radiographic surveillance to detect screw migration before loss of correction occurs.

## 10. Comparison with Soft Tissue Release Alone

The role of combining proximal femoral guided growth with soft tissue release versus performing soft tissue release alone has been examined to determine the added benefit of the guided growth component. Sheu and colleagues conducted the first comparative study specifically addressing this question, comparing patients with cerebral palsy who underwent soft tissue release alone (64 hips) versus soft tissue release plus guided growth (24 hips) for hip displacement [20]. Although the guided growth group had more severe hip displacement preoperatively, they demonstrated a significantly greater 2-year decrease in migration percentage (−14.8% versus −11.8%). Among patients with a preoperative migration percentage greater than 50%, the rate of achieving a migration percentage less than 40% was greater in the guided growth group (73%) compared to the soft tissue release alone group (41%). Revision surgeries, mainly repeated guided growth and soft tissue release, were comparable between the groups. The authors concluded that adding guided growth to soft tissue release resulted in greater improvements in hip displacement than soft tissue release alone, with nonambulatory patients or those with severe hip displacement (migration percentage 50–70%) potentially benefiting from this less aggressive surgery by controlling migration percentage under 40% without requiring femoral osteotomy.

This comparative evidence supports the concept that guided growth provides additional benefit beyond soft tissue release alone in addressing the bony deformity component of hip displacement. The immediate improvement in migration percentage following soft tissue release, as noted by Lee and colleagues, addresses the dynamic soft tissue contractures, while the progressive improvement over subsequent months to years reflects the gradual varus correction achieved through guided growth [13]. This dual mechanism of action—immediate soft tissue balancing combined with gradual bony correction—represents a comprehensive approach to the multifactorial pathology of hip displacement in cerebral palsy.

## 11. Role in the Treatment Algorithm

Proximal femoral guided growth has emerged as an intermediate option in the treatment algorithm for hip displacement in cerebral palsy, positioned between soft tissue release alone and major reconstructive procedures, including osteotomy. For children with early hip displacement (migration percentage 30–50%) and significant coxa valga, guided growth combined with soft tissue release offers a less invasive alternative to immediate osteotomy while providing superior outcomes compared to soft tissue release alone. This approach aligns with the principle of using the least invasive effective intervention, particularly in young children with substantial remaining growth potential. However, one must be cautious and counsel patients and families clearly, given the reoperation rate of this procedure due to growing off the screw. Regular radiographic follow-up is required, although there is no widely accepted protocol currently published on this.

The ongoing GUIDANCE study, a multicenter randomized controlled trial comparing adductor-psoas tenotomy alone versus adductor-psoas tenotomy plus temporary medial hemiepiphysiodesis in children aged 2 to 8 years with GMFCS IV–V and migration percentage 30–50%, will provide important Level I evidence regarding the efficacy of adding guided growth to soft tissue release [21]. The study’s primary outcome of treatment failure at 5-year follow-up, along with secondary outcomes including patient-reported outcome measures, range of motion, and three-dimensional morphological changes to the proximal femur, will help clarify the role of guided growth in the treatment algorithm and identify predictors of treatment success and failure. However, even with this evidence, it is likely that many questions will still remain.

For children who have already undergone hip reconstruction, guided growth may serve a role in maintaining correction or addressing recurrent deformity, though the evidence suggests more modest improvements in this population compared to primary procedures. Zakrzewski and colleagues found that hips treated with guided growth after previous hip reconstruction showed improvements in neck–shaft angle and head–shaft angle, though migration percentage remained unchanged, suggesting that guided growth may help maintain bony alignment even when it cannot reverse established subluxation in the setting of prior surgery [17].

## 12. Current Evidence Quality and Future Directions

The current evidence base for proximal femoral guided growth in cerebral palsy consists primarily of retrospective case series and comparative studies, representing Level III and IV evidence. While these studies have consistently demonstrated radiographic improvements and acceptable complication profiles, the lack of randomized controlled trials limits the strength of recommendations that can be made. The GUIDANCE study represents an important step toward higher-quality evidence, and its results will be eagerly anticipated by the pediatric orthopedic community.

Several areas warrant further investigation. Long-term outcomes beyond skeletal maturity, including hip joint health, pain, function, and quality of life in adulthood, remain unknown. Specifically, the effect that this procedure has on the long-term shape of the proximal femur has yet to be described. It has been described that early physeal closure may occur. Thus, potentially decreasing the length of the neck. Additionally, the overall effect of the shape of the femoral head may have an impact on future clinical outcomes that have yet to be described. The optimal timing of screw removal to balance adequate correction with minimizing the risk of premature physeal closure and femoral head deformity requires further study. The role of guided growth in different subtypes of cerebral palsy (spastic, dyskinetic, ataxic) and the influence of motor distribution (hemiplegia, diplegia, quadriplegia) on outcomes have not been thoroughly examined. Cost-effectiveness analyses comparing guided growth to alternative treatment strategies would provide valuable information for healthcare systems and families.

Technical refinements, including optimal screw size, thread design, and placement techniques, continue to evolve. Some investigators have explored the use of multiple screws or alternative implants such as plates or staples, though the single transphyseal screw technique remains most commonly reported. The development of predictive models incorporating multiple patient and radiographic factors to identify ideal candidates for guided growth would enhance patient selection and counseling.

## 13. Conclusions

Proximal femoral guided growth, or temporary medial hemiepiphysiodesis, has emerged as a valuable minimally invasive treatment option for hip displacement associated with coxa valga in children with cerebral palsy. The accumulated evidence demonstrates consistent radiographic improvements in migration percentage, head–shaft angle, and other parameters of hip displacement, with a favorable safety profile compared to traditional osteotomy procedures. The technique is most effective in children with a migration percentage less than 50%, adequate remaining growth potential, and when combined with appropriate soft tissue releases to address dynamic muscle imbalances.

The procedure’s main limitation is the frequent need for screw exchange or reinsertion as the physis grows off the screw, though this represents a relatively minor intervention. Long-term concerns, including premature physeal closure and potential femoral head deformity, require continued investigation and careful patient monitoring. Patient selection remains critical, with factors including age, severity of displacement, acetabular dysplasia, and functional level all influencing outcomes.

As the evidence base continues to mature, particularly with the completion of ongoing randomized controlled trials, the role of proximal femoral guided growth in the treatment algorithm for hip displacement in cerebral palsy will become better defined. Current evidence cautiously supports its use as an intermediate option between soft tissue release alone and major reconstructive surgery, offering a less invasive approach that can delay or potentially obviate the need for more extensive procedures in appropriately selected patients. For the pediatric orthopedic surgeon managing children with cerebral palsy and hip displacement, proximal femoral guided growth represents an important tool in the therapeutic armamentarium, complementing rather than replacing traditional approaches and expanding the options available to optimize outcomes for this vulnerable population. Surgeons should, however, exercise caution when considering this option, given the lack of long-term evidence described. Therefore, a full safety profile of the technique cannot be completely understood at this time.

## Data Availability

No new data were created or analyzed in this study. Data sharing is not applicable to this article.
